# Genomic insight on *Klebsiella variicola* isolated from wastewater treatment plant has uncovered a novel bacteriophage

**DOI:** 10.1186/s12864-024-10906-x

**Published:** 2024-10-22

**Authors:** Kgaugelo E. Lekota, Refilwe O. Mabeo, Tsepo Ramatla, Deidre A. B. Van Wyk, Oriel Thekisoe, Lesego G. Molale-Tom, Cornelius C. Bezuidenhout

**Affiliations:** Unit for Environmental Sciences and Management, North-West University, Potchefstroom, South Africa

**Keywords:** *Klebsiella variicola*, Whole genome sequencing, Antibiotic resistant, Bacteriophages

## Abstract

*Klebsiella variicola* is considered an emerging pathogen, which may colonize a variety of hosts, including environmental sources. *Klebsiella variicola* investigated in this study was obtained from an influent wastewater treatment plant in the North-West Province, South Africa. Whole genome sequencing was conducted to unravel the genetic diversity and antibiotic resistance patterns of *K*. *variicola*. Whole genome core SNP phylogeny was employed on publicly available 170 genomes. Furthermore, capsule types and antibiotic resistance genes, particularly beta-lactamase and carbapenems genes were investigated from the compared genomes. A 38 099 bp bacteriophage was uncovered alongside with *K. variicola* genome. Whole genome sequencing revealed that the extended beta-lactamase *bla*_LEN_ (75.3%) of the beta-lactamase is dominant among compared *K. variicola* strains. The identified IncF plasmid AA035 confers resistance genes of metal and heat element subtypes, i.e., silver, copper, and tellurium. The capsule type KL107-D1 is a predominant capsule type present in 88.2% of the compared *K. variicola* genomes. The phage was determined to be integrase-deficient consisting of a *fosB* gene associated with fosfomycin resistance and clusters with the Wbeta genus *Bacillus* phage group. In silico analysis showed that the phage genome interacts with *B. cereus* as opposed to *K. variicola* strain T2. The phage has anti-repressor proteins involved in the lysis-lysogeny decision. This phage will enhance our understanding of its impact on bacterial dissemination and how it may affect disease development and antibiotic resistance mechanisms in wastewater treatment plants. This study highlights the need for ongoing genomic epidemiological surveillance of environmental *K. variicola* isolates.

**Supplementary Information:**

The online version contains supplementary material available at 10.1186/s12864-024-10906-x.

## Introduction

*Klebsiella variicola* is a Gram-negative, facultative anaerobic, non-motile, and rod-shaped bacterium belonging to Enterobacterales [1]. It forms circular, convex, and mucoid colonies and grows at approximately 11– 41 °C. Recently, the taxonomy of the *K. pneumoniae* complex has expanded and comprises *K. pneumoniae*, *K. quasipneumoniae* subsp. *quasipneumoniae*, *K. quasipneumoniae* subsp. *similipneumoniae*, and *K. variicola*. The *K. variicola* is a versatile bacterium capable of colonizing different hosts such as plants, humans, insects, and animals [2]. The species is mainly associated with opportunistic infections, such as those of the bloodstream [3], urinary tract [4], respiratory tract [1], and neonatal outbreaks [5].

*Klebsiella variicola* is mainly associated with opportunistic infections and is frequently identified as *K. pneumoniae*. The *K. variicola* can be employed alone or in collaboration with other organisms in the treatment of wastewater, biodegradation, and bioremediation of polluted soil in environmental protection [1, 2]. Antibiotic resistance genes can also have an impact on *K. variicola* genotypes, which can be a limiting factor in the bioremediation process. The misidentification of *K. variicola* implies a wrong epidemiology result as well as incorrect attribution to *K. pneumoniae* as the etiology of some severe infections [6]. Treating *Klebsiella* infections gets challenging as it's known that some *Klebsiella* species become resistant to various antibiotics [7]. Within this context, antibiotic-resistant *Klebsiella* infections have been shown to respond well to bacteriophages, a class of viruses that specifically target bacteria [8]. However, this isn't always the case because phage-mediated transduction have the ability to disseminate antibiotic resistant genes (ARGs) [9].

Although the use of phages to combat *Klebsiella* spp. has been studied since the early 1900s, the growing issue of antibiotic resistance has made it more relevant in the recent past [10]. Better taxonomic placement and classification of recently found phages are made possible by comprehensive genomic data [11]. Whole genome sequencing (WGS) enabled comparative genomics, which can provide light on the origins and diversification of phages by revealing their evolutionary links and divergence. Average Nucleotide Identity (ANI) is frequently used to identify new bacteriophages at the species level [12]. An ANI threshold of about 95% is frequently employed to distinguish between different species of bacteriophages[13]. Phages with an ANI of 95% or more are typically categorised as belonging to the same species, but those with ANIs below this cutoff are classified as separate species [14]. Within the rapidly growing discipline of virology, where genomic data is becoming more and more available and essential for precise taxonomy, this approach offers a strong framework for categorising phages.

Currently, *K. variicola* is gaining recognition as a cause of several human infections [15] nevertheless, its virulence profile is also not fully characterized. However, one of the notable virulence factors of *Klebsiella* spp. is the capsule, a protective structure that surrounds the cell wall and protects against host immune response, adhesion to surfaces, and evasion of antimicrobial agents [16, 17]. The interest in the capsule as an evolutionary and pathogenicity marker of therapeutically relevant strains was sparked by a genomics-based population study of multidrug-resistant (MDR) *K. pneumoniae* [18, 19]. The genetic diversity of the capsular locus has been greatly illuminated by WGS-based techniques. Severe infections brought on by certain bacteria are known to be associated with the expression of virulence factors and capsular (K) types, primarily K1 and K2. The K antigen variation as well as variations in other surface polysaccharides such as the O antigen have historically been employed for *Klebsiella* capsule typing [20, 21].

The clinical significance of *K. variicola* infection is hidden by imprecise detection methods that underestimate its real prevalence [22]. However, several methods have been developed to correctly identify this species. Recently, huge efforts have been made to study *K. variicola*; however, the biological aspects of this species are still unclear [23]. Infections caused by *K. variicola* have been reported in humans worldwide [15], however, there are fewer reports on the *K. variicola* strains isolated from the water systems. Effective water treatment processes are designed to remove or inactivate harmful *Klebsiella* spp., including *K. variicola* to ensure the safety of the water supply. This comes with understanding the biology of *K. variicola* and the presence of ARGs as well as the epidemiology of this bacteria. Regular monitoring, water quality testing, and adherence to proper microbiological and molecular identification techniques are essential for preventing waterborne diseases and protecting public health [24]. The inaccurate identification of the members of the *K. pneumoniae* complex has limited the study of *K. variicola*, leaving gaps in knowledge and clinical implications within healthcare systems. The improper discharge of human and animal waste into surface waters from hospitals, wastewater treatment plants (WWTPs), aquaculture farms, and surface, as well as groundwater all contribute to the spread of antibiotic resistance [25]. The population of resistant bacteria in the receiving water is growing due to the existence of antibiotic resistance bacteria (ARB) and antibiotic residues, which may prevent the growth of susceptible bacteria [25, 26]. The general rates of mutation, recombination, and lateral gene transfer as well as bacteriophages may be increased by antibiotic pollutants, heavy metals, and even chlorination [25, 27]. This could attract more genes into the mobilome and resistome while also acting as a driver of bacterial evolution with potentially detrimental effects on human welfare [27]. This study was aimed at characterizing *K. variicola* using population genomic structure, ARGs, and virulence genes particularly on the capsule typing, as well as the association of mobile genetic elements i.e., plasmids and bacteriophages.

## Materials and methods

### Isolation and identification of *Klebsiella* species

Influent and effluent water samples were aseptically collected in sterile 1 L bottles from selected wastewater treatment plants (WWTPs) in the North-West Province, South Africa. Enumeration and isolation of *Klebsiella* species was achieved using dilution series plated on *Klebsiella* ChromoSelect agar (Merck, Germany) selective media incubated at 37 °C for 24 h. Purified isolates of *Klebsiella* species were streaked onto *Klebsiella* ChromoSelect agar and were incubated at 37 °C for 24 h. Isolates were inoculated into nutrient broth and incubated for 24 h at 37 °C in preparation for DNA extraction. DNA was extracted using a NucleoSpin® Microbial DNA extraction kit (Macherey–Nagel, USA) in accordance with the manufacturer's instructions. The *Klebsiella* isolates were isolated from influent and effluent collected from WWTP B in the North West Province.

*Klebsiella* isolates (*n* = 14) were inoculated into nutrient broth and incubated for 24 h at 37 °C in preparation for DNA extraction. DNA was extracted using a NucleoSpin® Microbial DNA extraction kit (Macherey–Nagel, USA) following the manufacturer's instructions. A NanoDrop Lite 1000 spectrophotometer (model: Thermo-Fisher Scientific, USA) was used to determine the concentration and purity of DNA, which was ultimately stored at -80 °C until further analysis. A PCR assay was conducted to amplify the *Klebsiella* spp. housekeeping *rpoB* gene using the following primers: *rpoBA* F—AACGGTGTGGTTACTGACG and *rpoB* R—TCTACGAAGTGGCCGTTTTC, which produces a 108 bp amplicon size [28]. The PCR reactions constituted 12.5 μL of AmpliTaq Gold® DNA Polymerase, 0.05 units/L Gold buffer, 930 mM Tris/HCl pH 8.05, 100 mM KCl, 0.4 mM of each dNTP, and 5 mM MgCl2] (New England Biolabs, USA), 8.5 μL of RNase-nuclease free PCR water, 1 μL of 10 μM each primer and 2 μL of template gDNA. The cycling consisted of an initial denaturation at 95 °C for 5 min, followed by 40 cycles of 95 °C for 40 s, 60 °C for 30 s and 72 °C for 40 s, and a final extension of 72 °C for 7 min using the ProFlex PCR System (Applied Biosystems, USA). The negative control was a DNA free template (nuclease-free water). To allow standardization, molecular weight markers of 1 Kb and 100 bp DNA (PROMEGA, Madison, WI, USA) were used to determine the size of the PCR amplicons. For product size confirmation and yield estimation, 5 µL of the PCR products were loaded onto 1% agarose gel stained with ethidium bromide and subjected to electrophoresis for 45 min at 80 V and visualized under UV light.

The 16S rRNA gene was amplified from the *Klebsiella* isolate using two commonly employed universal primers for bacterial identification, that is, the 27F and the 1492R primers [29]. The PCR assay was conducted in a total volume of 25 µL reaction mixture consisting of 12.5 µL of the 2X DreamTaq Green PCR Master Mix (4 mM MgCl2, and loading buffer and 0.4 mM each of dATP, dCTP, mM dGTP, mMdTTP) (ThermoFisher Scientific, USA), 10 µM of each primer, 2 µL of template DNA, and 8.5 µL nuclease-free water. PCR conditions were optimized as follows: Initial denaturation step at 96 °C for 4 min, followed by 30 cycles of denaturation at 94 °C for 30 s, annealing at 57 °C for 30 s, and extension at 72 °C for 1 min, and finally a single and final extension step at 72 °C for 10 min. The representative PCR products were cleaned up using ExoSAP-IT (ThermoScientific, USA), and subjected to cycle sequencing using the BigDye Terminator v3.1 kit (ThermoScientific, USA), and sequenced on the SeqStudio genetic analyzer at North-West University, Potchefstroom, South Africa.

### Antibiotic susceptibility test

Selected *Klebsiella* isolates were purified by streak plate method and, these were subjected to the Kirby-Bauer disk diffusion method to determine antibiotic resistance. Antibiotics used in this study were purchased from ThermoFisher ScientificTM (Johannesburg, South Africa) and are listed as follows: ampicillin 10 µg, amikacin 30 µg, cephazolin 30 µg, cefotaxime 30 µg, ceftriaxone 30 µg, cotrimoxazole 25 µg, imipenem 10 µg, gentamycin 10 μg, nitrofurantoin 300 µg, norfloxacin 10 µg, chloramphenicol 30 µg and ofloxacin 5 µg. Results from antibiotic resistance susceptibility tests were interpreted using Performance Standards for Antimicrobial Susceptibility Testing (2018) provided by the Clinical and Laboratory Standards Institute (CLSI) [30].

### Whole genome sequencing and bacterial genome assembly

DNA library preparation of the strain was executed using the rapid Barcoding Sequencing kit [SQK-RBK004] (Oxford Nanopore Technologies, United Kingdom) and performed according to the manufacturer’s instructions. Libraries comprising a *Klebsiella* isolate barcode were multiplexed, and further sequenced with qualified FLO-MIN106 flow cells (R9.4.1, active pore number ≥ 800) for 48 h on MINion MK1B sequencing platform (Oxford Nanopore Technologies, Oxford, United Kingdom). A Guppy basecaller 5.0.17 was utilized to convert raw data in fast5 format to the base called data in fastq format. Debarcoding of samples was performed with the same software together with the base calling procedure. All reads with the quality Q < 7.5 were excluded from the subsequent data analysis. The quality of trimmed data was assessed using NanoPlot v1.18.1 [31]. Quality filtering was performed using FiltLong v0.2.0 https://github.com/rrwick/Filtlong). The filtered ONT reads of strain T2 were de novo assembled with Flye v.2.3.3 [32] and polished using the Medaka (https://github.com/nanoporetech/medaka) consensus pipeline. CheckM [33] was used to assess the potential contaminants in individual assembled genomes. Quast v 2.3 [34] was used to evaluate the draft genome assemblies of the strain. The assembled contigs were annotated using NCBI prokaryotic genome automatic annotation pipeline (PGAAP) [35] and rapid annotation using subsystem technology (RAST) [36].

### In silico taxonomic classification and pangenomics analysis

In silico taxonomic classification of the bacterial strains was conducted using MLST [37] and rMLST [38]. All the retrieved and sequenced *K. variicola* genomes in this study were further annotated using Prokka v.1.14. [39]. Similarity searches between the coding domain sequences (CDSs) of assembled genomes were conducted using pair-wise BLASTp [40] and Markov Cluster Algorithm (MCL). Clusters were created using paralogs of the genomes and were ordered by the presence/absence of orthologs [41]. Pangenome clusters were defined as follows: Core-genes present in all isolates; soft core-genes present in at least 95% of isolates; shell-genes present between 15–95% of isolates; cloud-genes in less than 15% of isolates. The SNP-sites 2.5.1 (https://github.com/sanger-pathogens/snp-sites) [42] was used to filter the single nucleotide polymorphism on the core genome alignment. Gubbins [43] was used to identify and remove recombination within the fill alignment. Using the core SNPs found in all isolates, an ML phylogeny was created using IQ-TREE version 1.6.10. To visualize the matrix showing the presence and absence of core genes in the used strains, Phandango was used. The phylogenetic tree of the *K. variicola* genomes was visualized using ITOL [44].

### Antibiotic resistance, virulence gene detection, plasmid replicon determination

Kaptive [18] was used to determine the capsule types in the *K. variicola* genomes (*n* = 170). The ABRicate pipeline [assessed on 25 July 2023] and AMRFinderplus [45] were employed to identify antibiotic resistance and virulence genes in the genome of the *K. variicola* strain. Antimicrobial resistance determinants were identified in the assembled genome using the ResFinder database (–db ResFinder) with minimum identity and coverage thresholds of 75% (– minid 75) and 50% (–mincov 50), respectively. The Comprehensive Antibiotic Resistance Database (CARD) was also employed to determine the AR genes. ABRicate was further used to determine the efflux pump coding genes and virulence factors in the sequenced genome using the Virulence Factor Database [VFDB; –db vfdb] [46] using minimum identity and coverage thresholds of 70% (–minid 70) and 50% (–mincov 50), respectively. Plasmid replicons were identified by ABRicate on the sequenced genomes by using the Plasmid Finder database [47]. The MOB-Typer tool from MOB-Suite software v1.4.9 [48] was also used to characterize plasmid sequences. Circular plasmid maps were created using the Proksee server (https://proksee.ca). FastANI [49] was used to compare the plasmids of the closely related replicon. VirSorter [50] and MobileOG-db [51] were used to determine the mobile genetic elements.

### Phage genomic analysis

The quality and genome completeness of the phage was assessed using QUAST [34] and CheckV [52] using default parameters, respectively. Genome annotation was done with the Pharokka pipeline 1.2.1 [53], and screened for tRNAs, antimicrobial resistance genes, and virulence factors with tRNAscan-SE v2.0.11 [54], Comprehensive Antibiotic Resistance Database (CARD) [55], and Virulence Factor Database (VFDB) [46], respectively. The circular plots were also generated using Pharokka. To understand the relationships of the phages to previously characterized phages, a proteome-based phylogeny was constructed using the ViPTree [56] and subject to BLASTn and tBLASTn against NCBI. VICTOR [57] was used to determine the evolutionary relationships between whole-genome phages of the Bacillota host group, which utilises an optimised Genome Blast Distance Phylogeny (GBDP) to infer phylogenetic trees. The average nucleotide identity (ANI) of our phages was compared with the genomes identified using the Virus Intergenomic Distance Calculator (VIRIDIC) [58]. Genomes with an ANI > 95% were designated as the same species. The probabilities of each phage having virulent or lysogenic lifestyles were estimated using BACPHLIP [59]. Gene content and synteny among phage genomes were compared with Clinker [60] with a 50% similarity threshold. The phageTB server [61] was used to determine the interaction of the phage with the host bacteria.

### Determination of the *Bacillus* phage FI on *Klebsiella* species isolates

The presence of phage genes were determined among *Klebsiella* species isolates by using conventional PCR assay. Phage primers were designed using Primer3Plus tool [62]. This included three primer pairs (Supplementary table S1), that target three encoding sites i.e. Phage terminase large subunit, hypothetical proteins, and tail fiber spike protein regions. The PCR assay consisted of a total reaction of 25 μL containing 12.5 μL of a 2X DreamTaq Green Master Mix (0.4 mM dATP, 0.4 mM dCTP, 0.4 mM dGTP, 0.4 mM dTTP, 4 mM MgCl2, and loading buffer) (ThermoFisher Scientific, South Africa), 8.5 μL of nuclease-free water, 2.0 μL of the template DNA, and 1.0 μL of each oligonucleotide primer. PCR conditions were optimized as follows: Initial denaturation step at 95 °C for 3 min, followed by 30 cycles of denaturation at 95 °C for 30 s, annealing at 60 °C for 30 s, and extension at 72 °C for 1 min, and finally a single and final extension step at 72 °C for 10 min. PCR reactions were performed using the ProFlex PCR System (Applied Biosystems, USA).

### Accession numbers

The 16S rRNA sequence obtained in this study has been deposited to the GenBank database with assigned accession number (OR722220). The genome of *Klebsiella variicola* strain T2 has been assigned the following accession number: GenBank CP133153-CP133158, and the phage genome: OR487170 (Table 1).

**Table 1:** Genomic features of *Klebsiella variicola* and plasmid typing^a^

Replicon	Accession number	Size	GC	Plasmid replicon type(s)	ARGs	Relaxase type(s)
Chromosome	CP133153	5,683,738	57.4	-	*oqxA, oqxB*, *emrD, fosA, kdeA, bla*_LEN_	-
Phage	OR487170	38,099	35.1	*-*	*fosB*	-
Plasmid p_AC125	CP133154	77,302	48.7	IncFIB	-	-
Plasmid p_AA022	CP133155	124,711	53.0	IncFIB(pQil)_1_pQil, I IncFII_1_pKP91	-	MOBF
Plasmid p_AA439	CP133156	69,777	54.2	IncFII	-	MOBF
Plasmid p_AA035	CP133157	221,901	52.3	IncFIB(K)_1_Kpn3, IncFII_1_pKP91	-	MOBF
Plasmid unnamed	CP133158	64,563	54.3	IncR	-	MOBC

^a^All the plasmids had the match nucleotide BLAST percentage related to *K. pneumoniae*. (-) Indicates absent

## Results

### Identification of the *Klebsiella variicola*

The *Klebsiella* spp. strains were isolated using classical microbiological tests on ChromoSelect agar, which resulted in 14 suspected *Klebsiella* colonies. Amongst these isolates, 10 were retrieved from the influent, while 4 isolates from the effluent. A PCR assay using the *rpoB* housekeeping gene was used to establish the presence of 14 *Klebsiella* spp. The *K. variicola* isolate was further confirmed by 16S rRNA gene sequencing. The query sequence of the *K. variicola* isolates sequenced shared high (99.97%) similarity with *K. variicola* strains KKP012 (CP088956.1) and strain AHKv-S01 (CP047360.1) available in GenBank.

All isolates were further subjected to antimicrobial susceptibility test to evaluate their resistance patterns. All the isolates were resistant to ampicillin, and 8 (57.1%) isolates to Kanamycin (Supplementary Table S1). The isolate INF- 2A (*K. variicola* strain T2) was resistant to more than 3 classes of antibiotics, which included Cephalosporin (Cephazolin), aminoglycoside (Ampicillin), Quinolone (Norfloxacin).

### In silico taxonomic classification

The number of sequenced reads generated from the *K. variicola* strain after filtering was 37,596 with an average read length of 9560.5 bp. The quality score of sequence reads that passed at > Q5 and Q7 were 100%, while at > Q10 and Q12 was 68.1% and 17.6%, respectively. Sequence reads of *K. variicola* were assembled into 6 contigs that resulted in a genome size of 6 177 429 bp with an average GC content of 53.3%. This genome consists of 6 342 coding sequences (CDSs) with 133 total number of RNA genes. The use of Genome Blast Distance Phylogenies (GBDP) assigned this genome as *K. variicola*. Genetic features of the *K. variicola* show that the sequenced genome strain comprises of 6 assembled replicons that include 1 chromosome, 4 plasmids and 1 phage (Table 1). The PubMLST identified the sequenced genome as 100% identical to *K. variicola*. Ribosomal MLST in silico analysis identified the sequenced genome as 67,031, which is most closely related to the human isolate genomes YD626-2 (GCA002886665.1: USA) and 171J7 (GCA008375025.1: France).

### Pangenome analysis of *K. variicola*

A pan-genome of 170 K*. variicola* strains was constructed to assess genetic diversity using core and accessory genes. High proportions of *K. variicola* genomes compared in this study were isolated from human (*n* = 150), followed by animals (*n* = 10), plants (*n* = 5), and water (*n* = 3) sources. The sequenced and compared global *K*. v*ariicola* showed evidence of distinct genetic diversity clustering the sequenced genome with GCA_002886665.1 and GCA_008375025 genomes that were both isolated from humans (Supplementary Figure S2). Average nucleotide identity (ANI) shows that *K. variicola* strain T2 is 99.49% similar to the France strain 171J7 (GCA_008375025). In this study, the pangenome was defined by 36 403 genes determined across the compared *K. variicola* genomes. There were 2194 core genes of the *K. variicola* identified, whereas the shell and cloud genes were 2833 and 29,844, respectively. The genome of *K. variicola* strain T2 represented most of the genes annotated as hypothetical proteins or pseudogenes (*n* = 3216) due to the presence of INDELs and detected prophages. This was observed in the gene cluster that shares unique core genes that mostly constitute hypothetical proteins (Supplementary Figure S3). The use of ribosomal-MLST corresponds with the pangenome placement of the sequenced genome, which grouped it with GCA_002886665.1 and GCA_008375025 strains of the USA and France, respectively. However, genome T2 has accessory genes that mostly include hypothetical proteins (Supplementary Figure S4). Meanwhile, other genes include HTH-type transcriptional regulator Tfds, Periplasmic murein peptide-binding protein MppA_2, Ferrienterobactin receptor fepA_5, Type IV pilus biogenesis and competence protein PilQ. The core SNP phylogenetic tree showed that the sequenced genome shows a distinct lineage that is presented with a high number of 1204 SNPs, which groups closely with the genomes of GCA0224744351 and 171J7 strains. The three-compared genomes constituted of 1 407 SNPs, meanwhile 413 SNPs were assigned as core SNPs that grouped them in one sub-clade. High number of SNPs inside recombinations (*n* = 963) were found as opposed to SNPs outside recombinations (*n* = 241) on the sequenced *K. variicola* genome strain T2.

### Antibiotic resistance, stress response genes and virulence genes in *K. variicola* strain T2

The chromosomal genome consisted of genes *oqxAB*, *fosA5*, and *bla*_LEN_ that confer resistance to phenicol/quinolone, fosfomycin, and beta-lactam, respectively (Supplementary Table S3). The multidrug efflux MFS transporter *emrD* gene was also identified in the chromosome. The plasmid AA035 which is about 221,901 bp (320 CDSs) conferred genes for resistance to metal resistance i.e., silver (*sil-ERCBAP* gene cluster), copper (*pco-BCDRS* gene cluster), and tellurium (*ter-DCB* gene cluster). The heat resistance system genes were also identified in this plasmid (i.e. *shsP*, *yfdx1*, *yfdx2*, *hdeD-GI*, *trxLHR*, *kefB-GI*, and *psi-Gi*). Moreover, the RAST annotation identified genes involved in stress response gene such as *gfa*, as well as proteins HMG-DH and FGH, which are associated with the glutathione-dependent pathway of formaldehyde detoxification. Additionally, the annotation revealed 82 genes related to the IncF conjugal transfer system. Notably, none of the plasmids were found to carry antibiotic resistance genes. Among the plasmid CP056308 shares genetic features with the sequenced *K. variicola* plasmid AA035, consisting of the silver gene cluster (s*il-ESRCFBAP*), copper gene cluster (*pco-ABCDRS*), and heat resistance system (*shsP, yfdx1, yfdx2, hdeD-GI, trxLHR, kefB-GI* and *psi-Gi*). The tellurium (*terDCB* gene cluster) was exclusive to the *K. variicola* strain T2 IncF plasmid AA035. The type 1 fimbriae (*fimEA*), outer membrane protein A (*ompA*), the phenolate siderophore enterobactin (*entAB*), ferrienterobactin ABC transporter (*fepC*), and classical *K. pneumoniae* virulence factors were identified in the *K. variicola* strain T2. This strain also contained several genes from the type VI secretion system (T6SS) or *tran/trap* gene clusters.

### Beta-lactamase genes and capsule typing

In this study, the 170 K*. variicola* genomes that are publicly available, including the sequenced genome, were examined for the presence of beta-lactamase genes. The strains were made up of one or more *bla*-genes. Among the examined *K. variicola* strains, the *bla*_LEN_ gene type is the most common (75.3%). The *bl*a_LEN-2_ gene found in the sequenced strain in this study can be found in 31 of the compared genomes. Other different *bla*_LEN_ genes were noticeable that included *bla*_LEN-16_ (*n* = 31), *bla*_LEN-17_ (*n* = 10), *bla*_LEN-19_ (*n* = 1), *bla*_LEN-2_ (*n* = 32), *bla*_LEN-21_ (*n* = 3), *bla*_LEN-22_ (n = 5) and *bla*_LEN-24_ (*n* = 42). The *bla*_CTX-M-15_ (*n* = 37) is the second most prevalent gene found in the compared strains. Despite the presence of *bla*_LEN_ and *bla*_CTM-M_ genes, strain KPN2043 (GCA_021897435.1) consisted of 5 different *bla*-genes (*bla*_CTX-M-15_, *bla*_LEN-24_, *bla*_NDM_, *bla*_OXA_, *bla*_TEM-1B_) isolated in 2020 from a clinical host in Melborune, Australia. All the compared water sources isolates consisted of the *bla*_LEN_ gene profile, meanwhile, all clinical isolates show diverse *bla*-genes including the carbapenemase *bla*_KPC-2_ and *bla*_KPC-3_ genes.

**Fig. 1:**
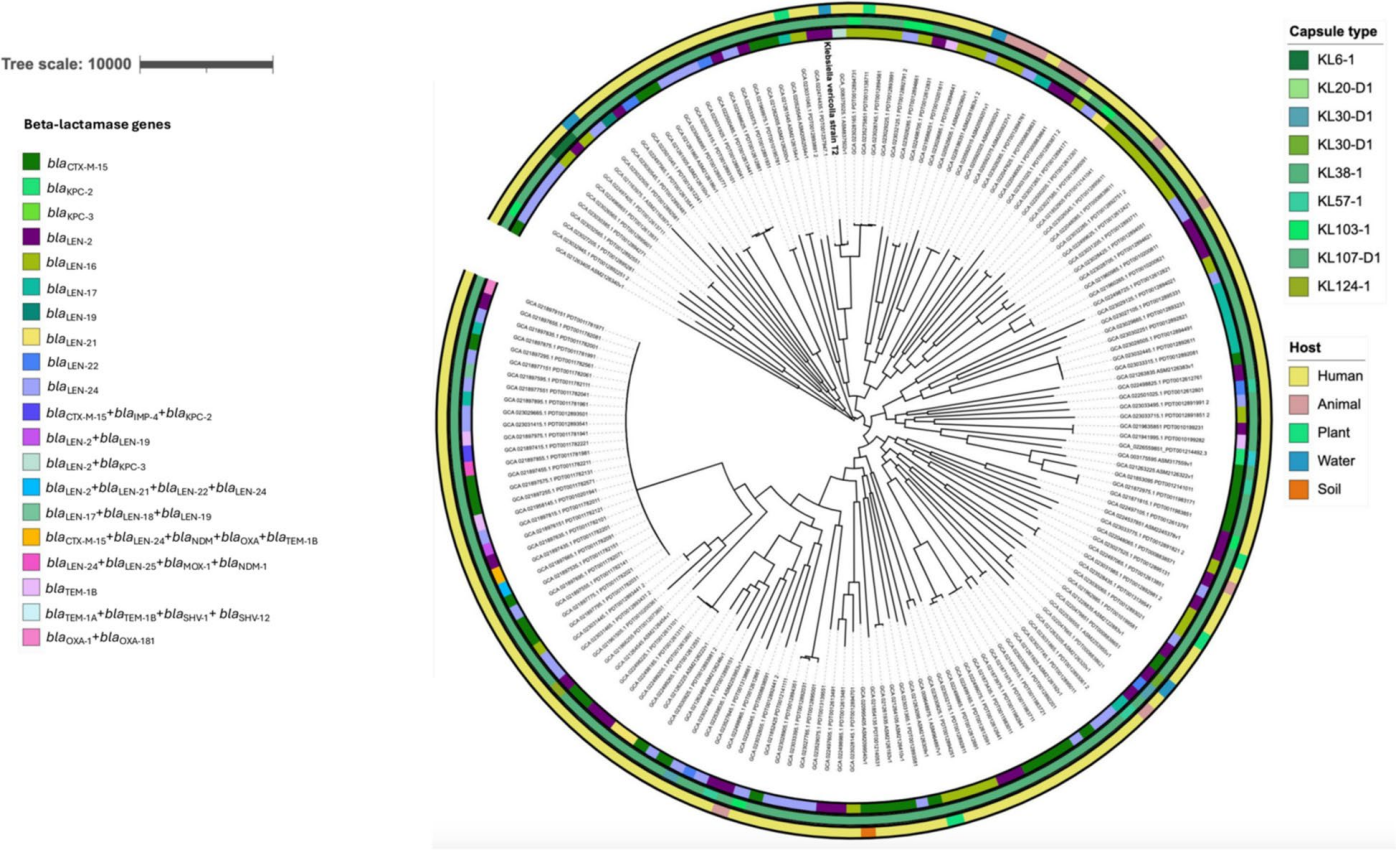
Maximum likelihood phylogeny using core SNP identified among 170 *Klebsiella variicola* genomes that includes the sequenced genome, highlighted in bold black. Host and capsule types are colour coded in their different respective clusters. The first inner circle represents the host of the *K. variicola* strains and the second outer circle represents the capsule types identified in this study

Capsule prediction was determined among the 170 genomes including the sequenced *K. variicola* strain. This study reveals that the KL107-D1 capsular serotype, identified within this sequenced genome, predominates among *K. variicola* strains (*n* = 150). Seven genomes present a KL103-1 capsule type, while two genomes had a KL57-1 capsule, as well as two genomes with a KL30-D1 capsule type profile. Figure 1 displays other various capsule types (*n* = 1) that are less frequently observed in other compared genomes.

### Plasmid replicon types

Five plasmid replicons were identified in the genome of *K. variicola* strain T2 without antibiotic resistance genes. All the plasmid replicon type match identifications are related to *K. pneumoniae*. Their sizes ranged from 64 653 bp to 221 901 bp with a GC content of 48.7 to 53.4% (Table 1). Different IncFIB replicon types were identified in the three identified plasmids AC125, AA022, and AA035, while replicon type IncFII was found in plasmids AA022 and AA439. The mobilization (MOB) type plasmids which consisted of the *tra*/*tnp* gene clusters were found across the 3 plasmids, except in plasmid AC125 (Supplementary Figure S4). The conjugative plasmids MOBF were found in the three plasmids namely AA022, AA439, and AA035. None of the transposons or insertion genes were found in the plasmid AC125. This plasmid is similar to strain T877 plasmid pKPT877 (Accession no. CP0842431) and shows high synteny with the aligned plasmid shared by 25 orthologous genes. Comparison with the related *Klebsiella* spp. plasmid shows that the *K. variicola* plasmid AA035 ANI is 96.2% similar to *K. pneumoniae* plasmid pRHBSTW − 00832_2 (CP056308) that is 232 875 bp isolated from freshwater sample downstream of a wastewater treatment plant in the United Kingdom. The gene organisation/ synteny of the two compared plasmids share about 53 orthologous genes. Plasmid AA022 is 99.66% similar to the *K. pneumoniae* strain E16KP0218 plasmid PE16KP0218-1 (Accession no. CP052287.1) at 80% coverage. This plasmid consists of IncF conjugal transfer (*n* = 62) genes, as well as *cynS* and *cynT* genes that encode cyanide dehydratase. The mobile genetic elements IS*66* family transposase (IS*Ec22* and IS*cfr14*), IS*3* family transposase (IS*Kpn38*, and IS*Yps8*), IS*NCY* family transposase IS*Bcn27*, and IS5 family transposase IS*Kpn26* are found in this latter mentioned plasmid. The plasmid AA439 is 96.77% similar to the *K. pneumoniae* strain C16KP0108 plasmid pC16KP0108-4 (Accession no. CP052438) with 95.4% ANI at 90% coverage (19/21 orthologous match). Multiple sequence alignment of these two plasmids resembles the translocation of genes.

### Determination of prophage regions on the chromosome

The *K. variicola* chromosome contained five prophages, with sequence lengths ranging from 77 743 base pairs to 222 800 bp and an average GC content of 54% (Supplementary Figure S4). The Pantoea phage PdC23 is somehow connected to the prophages 0, 2, and 4. Subsequently, listed phages exhibit significant substitutional nucleotide differences that range from 0.05 to 0.5. According to PHASTER analysis, phage region 0 resembles phage *Edwardsiella* GF 2, which was found in tissue homogenates of a cultured Japanese flounder (*Paralichthys olivaceus*) that died from edwardsiellosis in Japan (NC026611). Since both are lysogenic, phage *Edwardsiella* GF 2 and the sequenced prophage region 0 share the same lifestyle type. In addition to the prophage regions, a non-integrase *Bacillus* phage FI was sequenced coccurently with the *K. variicola* strain T2 during whole genome sequencing. Using in silico analysis, this phage is absent on the chromosome of *K. variicola* strain T2. The use of PCR assay using the the designed *Bacillus* phage FI primers, showed that this phage is absent among the other tested 13 *Klebsiella* isolates.

### Genome features of the non-integrase *Bacillus* phage FI

**Fig. 2:**
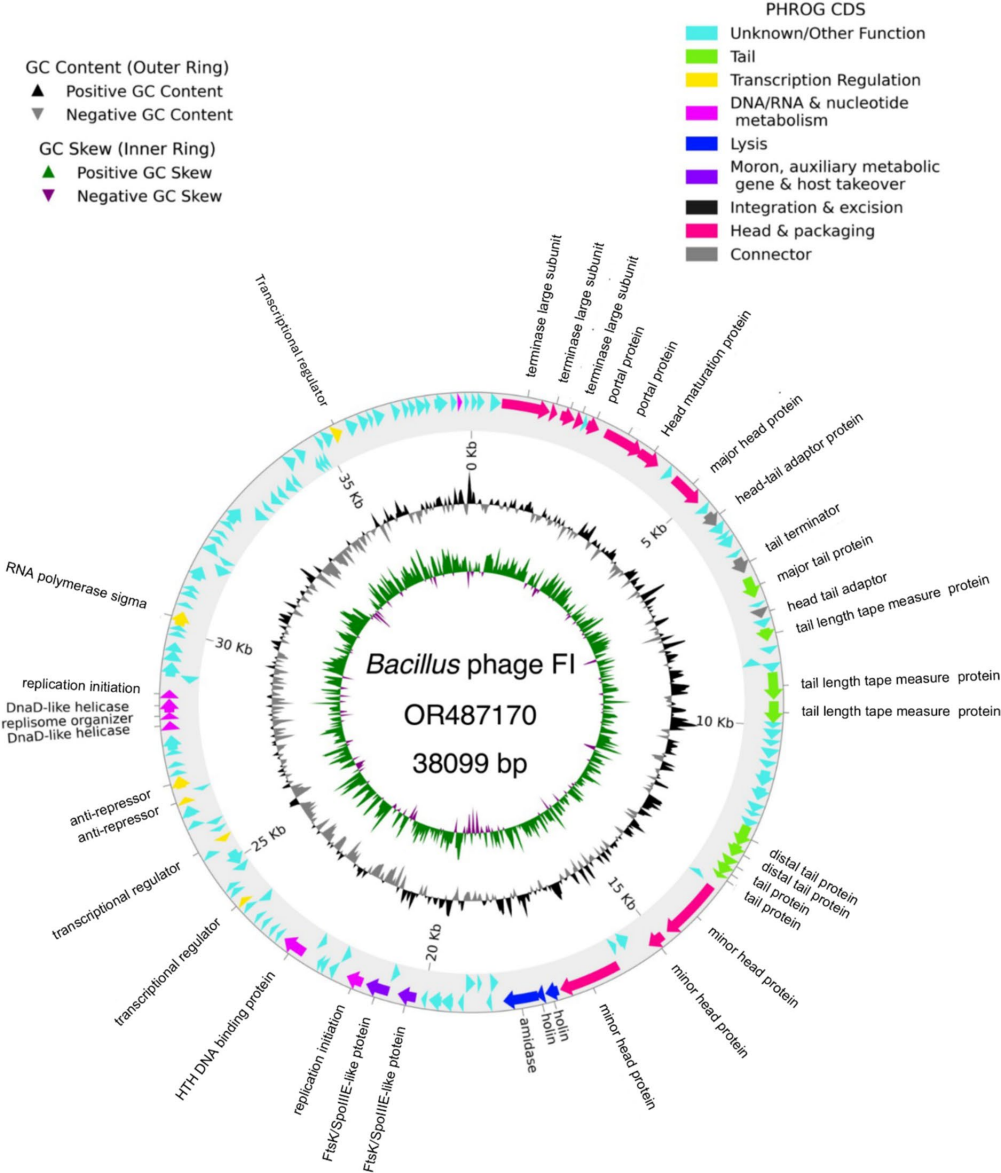
Complete genome structure of the *Bacillus* phage FI illustrating its PHROG coding sequences and a genome size of 38,099 bp. Outside the circle is the annotation of the genes with known functions listed on the map. The GC skew is presented in the inner circle followed by GC content

The *Bacillus* phage FI with genome size of 38,099 bp (Fig. 2) was found within the sequenced bacterial genome. Most temperate and virulent *Klebsiella* host phages are characterized as having a high GC content of between 39–57%. However, the identified *Bacillus* phage FI genome has a GC content of 35.06% consisting of two anti-repressors and proteins (amidase and holins) involved in the lysis-lysogeny decision of temperate phage. The phage was classified as a temperate phage, and Pharokka annotation using the top hits matched this genome with *Bacillus* phage Gamma (DQ222853) with a 0.00940624 mash distance. *Bacillus* phage Gamma is classified as belonging to a class of Caudoviricetes, phylum Uroviricota, under the Wbeta virus genus with a GC content of 35.221%, which is closely related to the *Bacillus* Gamma or Cherry phages. The phage consists of a higher number (*n* = 145) of coding sequences (CDS) as compared to *Bacillus* phage Gamma, and with no integrase or excision genes found in this genome (Supplementary Table S4). About 71,7% (*n* = 104) of the genes were classified as unknown genes (Supplementary Table S4). The phage consisted of a *fosB* gene associated with fosfomycin resistance which was detected using AMRFinderPlus.

### Placement of the *Bacillus* phage Fi and host determination

**Fig. 3:**
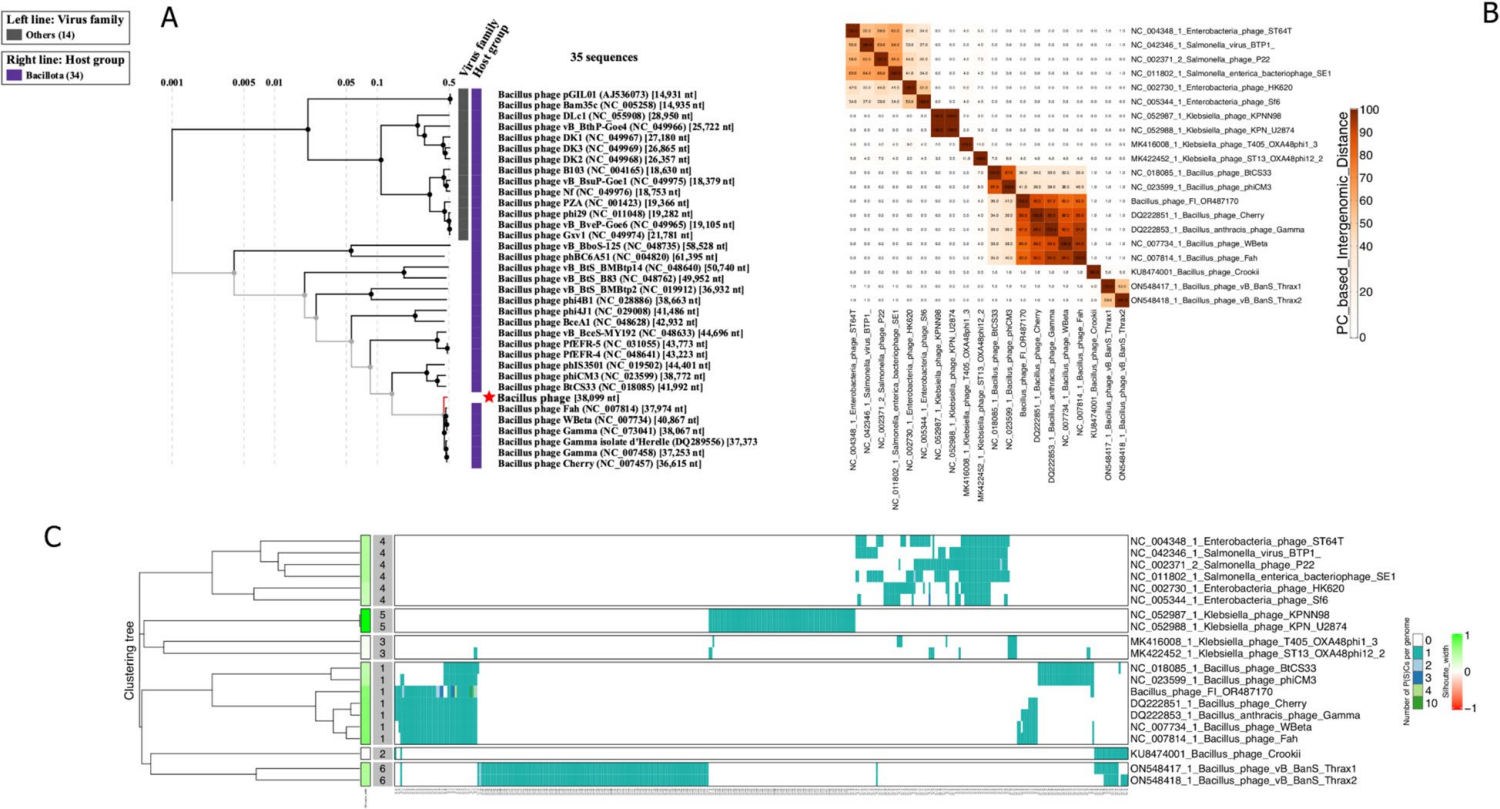
Proteome-based phylogenetic relationships and heatmap clustering of the Bacillota group phages with the placement of the sequenced phage in this study. **A** Present clustering of the phages among different host groups with virus family name, placing the *Bacillus* phages as *Wbeta* genus of the Bacillota group. **B** A protein similarity heatmap between the 20 phages showing the similarities of the *Bacillus* phage FI with *B. anthracis* phages (based on ≥ 70% intergenomic similarity to cluster). The inclusion of Enterobacteria and *Salmonella* phages was based on Enterobacteriaceae host group to show the relatedness of the sequenced phage. Intergenomic similarities (scale from 0–100%) are shown on the right. **C** Genome clustering protein heatmap of the Bacillota group and Enterobacteriaceae phages

*Bacillus* phage FI represents a novel phage of *Klebsiella* that groups among Bacillota host group of phages (Fig. 3). *Bacillus* phages of *B. anthracis*, such as phage Gamma, cherry, Wbeta, and Fah, are fed by the Bacillota host group (Fig. 3A). Based on BLASTn searches against the non-redundant database, the *Bacillus* phage FI genome in the current investigation displayed a nucleotide identity value of less than 95% similarity in relation to deposited *Bacillus* phage *Wbeta* genus viruses. The inclusion of some of the *Klebsiella* phages (*n* = 4) and *Salmonella*/ Enterobacteria phages (*n* = 6) was based on close relationship identification by PhageAI, which were identified as belonging to the Siphoviridae phages under the genus of *Lederbergvirus* (Fig. 3B). However, there is inconsistent grouping as they are distinctly related to the identified phage in this study. Analysis using VIRIDIC revealed the same grouping clustering the *Bacillus* phage FI into the same genus species of Wbeta group (≥ 70%) intergenomic similarity (Supplementary Figure S5). Intergenomic nucleotide similarity ranged from as high as 82% to 90%, just below the intra-species boundary (> 95%) (Supplementary Fig. 5). However, the intergenomic protein similarity between *Bacillus* phage FI and *Bacillus* phage cherry was 90% (Fig. 3B). Despite *Bacillus* phage FI grouping closely with these phages, based on protein homology, it displays a distinct subgroup among the compared Bacillota host phages (Fig. 3C). Genome clustering protein grouped the Bacillota phages with *Bacillus* phage FI belonging to cluster 1. This cluster mainly caters *B. anthracis* host group, meanwhile, other unique *B. anthracis* host group cluster 2 of the South African *Bacillus* phage crookii was determined [11]. The use of VICTOR analysis using whole genome nucleotide sequences also groups this phage with the *Bacillus* phages, with an average genomic size of about 38 kb and a 35% GC content (Supplementary Figure S6). It is also evident that *Bacillus* phage FI forms a distinct species that belongs to the same genus of Wbeta virus. Comparative host interaction of the phage showed that *Bacillus* phage FI interacts with the *B. cereus* genome, as opposed to *K. variicola* strain T2.

### The virion morphogenesis, tail and lysis module

**Fig. 4:**
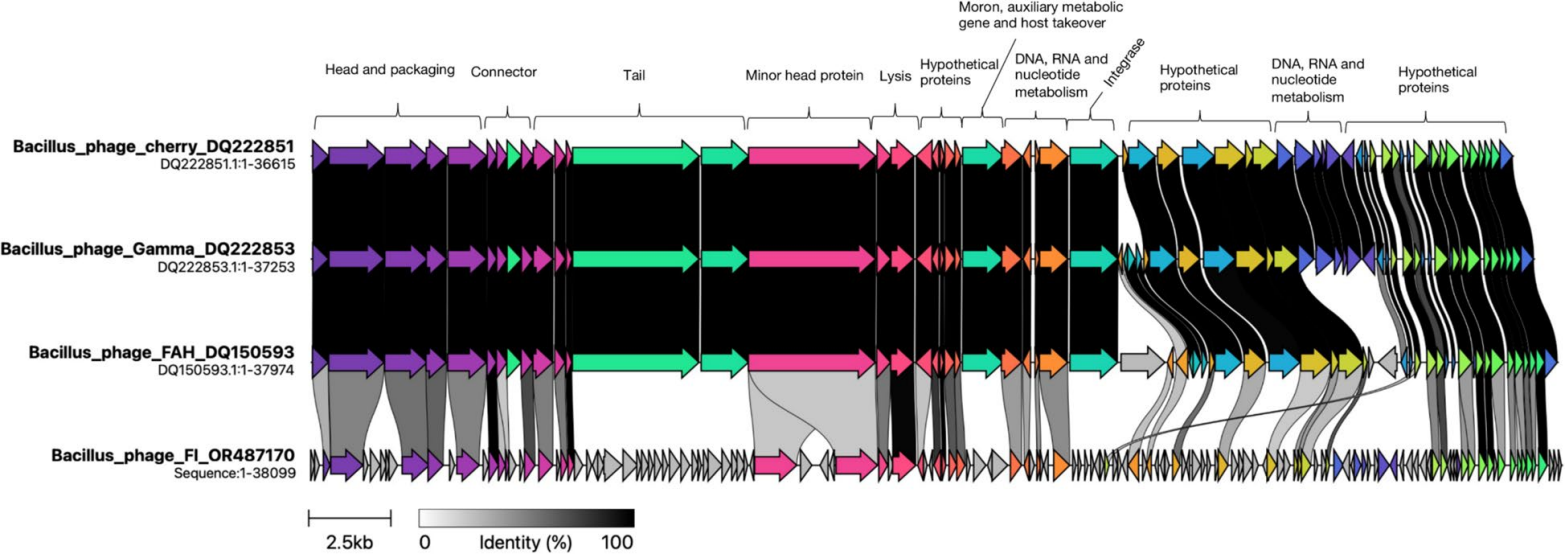
The virion morphogenesis, tail and lysis module in the *Bacillus* phage FI and compared lytic *Bacillus* phages. Gene synteny and identity shown by black and greyscale shading, shows that *Bacillus* phages are quite similar as compared to the *Bacillus* phage FI. Module alignment of the head and packing that include large terminase subunits on *Bacillus* phage FI is 59% similar with the two compared *Bacillus* phages. The minor head protein of the two *Bacillus* phages are less conserved (39.5%) when compared to the *Bacillus* phage FI. Annotation using Pharokka assigned *Bacillus* phage FI with no antibiotic resistance and virulence factors

Due to the absence of integrase in *Bacillus* phage FI, the *Bacillus* phages cherry, FAH and gamma are related and are regarded as lytic phages. Despite coming from different bacterial host species, the synteny map between the *Bacillus* phages and the sequenced *Bacillus* phage FI (Fig. 4) shows that their genomes share a high degree of similarity in terms of gene content and orientation, with only a small number of genes occurring in their subset. However, the sequenced *Bacillus* phage FI has a significant number of CDS, with 145 counts (Fig. 4). Phage FI shares several similarities with the two lytic phages in terms of genes like the larger terminase subunits of the head and packaging i.e. 59% similarities encoded in the same direction. The small terminase subunits of phage FI is less conserved with 39.5% similarity to *Bacillus* phages. In contrast to the two known lytic phages, *Bacillus* phage FI contains a higher number of head and packing genes (*n* = 11). One connector gene between the head and tail packaging and lytic phages is 100% identical. One connector gene of the head and tail packaging of the *Bacillus* phage FI is 100% identical to the lytic *Bacillus* phages. The *Bacillus* phage FI tail gene cluster is completely different from the compared closely related phages. As a result, Phage FI shares a significant number (*n* = 9) of tail genes that are exclusive to the compared phage genomes. The two *Bacillus* phages have 100% identity with the *Bacillus* phage FI holin lysis gene. However, *Bacillus* phage FI revealed an endolysin gene that is absent from the compared *Bacillus* phages. In contrast to the studied *Bacillus* phages, *Bacillus* phage FI has two moron, auxiliary metabolic genes in comparison to *Bacillus* phages, *Bacillus* phage FI represents a higher number of putative proteins (*n* = 104).

## Discussion

This study isolated and sequenced a complete genome of *K. variicola* strain T2 concurrently with a *Bacillus* phage isolated from the influent of WWTP B in the North West Province, South Africa. Comparative genomics was carried out to understand the phylogenomic structure of *K. variicola*, determinants of antibiotic resistance, and virulence profiles of the sequenced genome. Furthermore, a novel non-integrase *Bacillus* phage was identified and has been characterised to understand its complexity. The sequenced *K. variicola* genome in the current study presents a new genotype with a high number of orthologous clusters that are mostly annotated as hypothetical observed among compared closely related genomes. An IncFB plasmid AA125 was also found in this study that relates to heavy metal stress or heat response in the WWTP. In South Africa, *K. variicola* bacteria are not well investigated particularly as *Klebsiella* complexes are known to be multi-drug resistant. Even if a microbiological test confirmed that this genome belonged to *K. pneumonia*, it is still necessary to identify and comprehend this strain's virulence factors. As a result, *K. pneumoniae* may have been incorrectly identified in numerous epidemiological studies on illnesses caused by *K. variicola* [22].

Knowledge regarding the genome content of *K. variicola* strains is important to provide insights regarding the plasticity of this species to acquire phenotypes responsible for its adaptation to different niches and hosts, focusing on virulence and AMR determinants. Therefore, this study also examined the virulence and antibiotic resistance genes profile in this genome. Furthermore, pangenome and wgSNP analysis was performed on the 170 global *K. variicola* strains. The examination of *K*. *variicola* strain T2 complete genome unveiled a chromosome, four plasmids, and a novel bacteriophage. Through the use of GDTB and MLST databases, this strain was unequivocally identified as *K*. *variicola*, belonging to sequence type 67,031. This sequence type, previously underexplored on a global scale, forms a distinctive cluster characterised by a notable abundance of hypothetical genes present in both the chromosome and plasmids. This revelation underscores the need for ongoing surveillance and investigation of *K*. *variicola's* presence in wastewater treatment plants, as inadequate management may pose potential health risks to the population. The study underscores the importance of comprehending the etiology and epidemiology of *K*. *variicola*, particularly in wastewater treatment plants, as South Africa has yet to report *K*. *variicola* cases stemming from such environments. Population genomic structure of *K. variicola* is now well established worldwide and there are no assigned clusters or single nucleotide polymorphisms to evaluate the transmission patterns of this bacteria. The use of phylogenomics and whole genome SNPs was determined in this study on the global strains (*n* = 170) that placed the sequenced strain with the human isolate genomes YD626-2 (GCA_002886665.1: USA) and 171J7 (GCA_008375025.1: France). The high number of SNPs determined on the sequenced genome indicates that the mining of *K. variicola* is not sufficient and is under-reported, particularly from WWTP.

It is quite evident that the use of MLST typing is not adequate for the typing of *K. variicola* strains. This has been determined in this study as most strains (*n* = 59) used in this study were unassigned and the sequence types do not correlate in the clustering of the identified *K. variicola* clades by whole genome phylogeny. This has also been reported in *K. variicola* genomes whereby 54% did not have ST assignation or had novel allele combinations (Long et al., 2017; Potter [4, 22, 63]. The use of MLST typing scheme for *K. variicola* (http://mlstkv.insp.mx) which is based on 7 housekeeping genes also showed that the sequenced genome in this study has a unique profile that could not be assigned in this scheme. Although *K. variicola* has been found in various natural niches [64], it is an important reservoir of antimicrobial-resistant genes (AMRs) such as extended-spectrum β-lactamases (ESBL), carbapenemases, and colistin resistance [4]. This study identified ARGs that are exclusively located in the chromosome of *K. variicola* genome. This included the *oqxAB*, *fosA5*, and *bla*_LEN_ genes that conferred resistance to phenicol/quinolone, fosfomycin, and beta-lactam, respectively. The phenicol/quinolone *fosA*, *oqxA* and *oqxB* genes have been reported in *K. pneumoniae* (*n* = 478) compared to genomes, located in the chromosome [65]. Meanwhile, the latter-mentioned genes were also found in *K. variicola* strains isolated from an urban river in South Korea [66]. The β-lactam *bla*_LEN_ have been reported to be mostly conserved among *K. variicola* genomes [4] and isolates recovered from health-care settings [67]. The gene was also present in the sequenced strain in this study, suggesting that the acquisition of this strain in contaminated water displays multiple antibiotic-resistance which may potentially affect humans.

One of the major virulence factors of *Klebsiella* spp. complex is the capsule polysaccharide (CPS) gene located on the chromosomal operon [16]. This study showed that the capsule KL107 is prominent on the compared genomes, including the sequenced genome in this study. The most common K-loci type KL107 have also been reported in *Klebsiella* spp. accounting for 67.9% of the compared strains, which have an association with *wzi* alleles 154 [20, 21]. The K-types are shared across *K. variicola* different sources as there were also other identified types in this study. Variation of capsule types among *K. variicola* genomes has also been found in other studies [22, 63]. However, prediction of capsule phenotypes is also recommended as genome data is complex as capsule formation is highly regulated and involves genes outside the K-locus region [68, 69].

Plasmids containing ARGs and heavy metal resistance have been reported in neonatal sepsis Turton [15], animals [70] and the environment [71]. In this study, the IncFIB plasmid AA035 found in the *K. variicola* strain presents characteristics of copper/silver and tellurium resistance. This plasmid is closely related to *K. pneumoniae* previously isolated from downstream of WWTP in the United Kingdom [72]. Since heavy metals in the environment are not degraded in WWTP, their presence could therefore represent a long-term selection pressure together with antibiotic resistance in *Klebsiella* species [71, 73]. Majority of the necessary proteins are encoded by the *tra*/*trb* genes cluster on conjugative plasmids, making conjugation a significant method of spreading virulence and antibiotic resistance among bacteria [74]. The *tra*/*trb* clusters and copper/silver genes, in particular, have been reported to be present on many, but not all, of the plasmids in clinical isolates of *K. variicola* and *K. pneumoniae*. This suggests that these genes could be easily transmitted in bacterial populations. The presence of heavy metal resistance genes may potentially lead to the spread of these plasmids among bacterial populations, and potentially contribute to the dissemination of metal resistance genes in the environment. The IncF and IncR type plasmids identified in this investigation carried no antibiotic-resistance genes and included individual IS mobile genetic components. Within a diverse array of *K. pneumoniae* and *K*. *variicola* genomes, it is not uncommon to encounter related plasmids that lack profiles of ARGs, as previously documented [60]. In this study, the other 4 identified plasmids mostly exhibit hypothetical genes, with the exception of plasmid AA022 that consists of the *cynT* and *cynR* genes that encodes carbonic anhydrases that catalyses the reaction of CO_2_ hydration to prevent the depletion of endogenous HCO3- levels due to rapid CO_2_ loss [75]. The carbonic anhydrases are found in all groups of microorganisms that are mostly chromosomal encoded. However, in this study the aforementioned genes were found in the plasmid.

It is often reported that the common trend of most bacterial host GC content correlates with their lytic or lysogenic phages [11]. This was also evident in the 5 identified prophages that were found in the sequenced bacterial genome in this study. However, this is not an absolute rule on lytic or lysogenic phages as GC variation has been reported in other bacterial hosts [76]. The host genome size of *K. variicola* is high at 6.2 Mb that is influenced by bigger sizes of the identified prophages and plasmids in this study. It has been previously reported that the GC content of bacteriophages tended to reduce with larger genome sizes [77]. In this study, the GC content of the prophages correlates with the *K. variicola* strain T2 genome. Whole protein phylogeny placement of the 5 identified prophages placed the sequences with the Pseudomonadota host phages. Based on PHASTER analysis, prophage 0 of the *K. variicola* strain T2 is somehow similar with phage *Edwardsiella* GF 2, which was found in tissue homogenates of a cultured Japanese flounder (*Paralichthys olivaceus*) that died from edwardsiellosis in Japan (NC026611) [76]. Bacteriophages can mediate the transfer of ARGs between bacteria via transduction, and a variety of antibiotic resistance genes (ARGs) have been identified in phage genomes in different soil samples [78]. *Bacillus* phage FI identified in this study was sequenced alongside with the *K. variicola* genome consisting of the *fosB* gene that confirms resistance to fosfomycin, this observation suggests that this phage can potentially aid in making the host to be resistant to fosfomycin. Therefore, it will also be important to understand the roles of phages in *K. variicola* and *Bacillus cereus* group at the WWTPs as they are under reported. The sequenced phage in this study is classified as integrase-deficient, meaning it lacks the integrase enzyme that is responsible for integrating the phage host DNA. Using the phageTB tool, this was also evident as there was no interaction of the phage genome analysis with the host *K. variicola* strain T2. The interaction is associated with the Bacillota group as also determined by the phage genome placement. The phage genome was determined as a temperate phage which has two anti-repressors and proteins (amidase and holins) involved in the lysis-lysogeny decision of the temperate phage. The absence of integrase in this phage genome has also been reported in other lysogenic phages consisting of many genes of anti-repressors, suggesting its involvement in controlling other regulatory proteins to convert from lysogenic to a lytic stage [79, 80]. Anti-repressors act as regulators that help to switch on the genes necessarily for the lytic cycle while preventing repressor proteins from inhibiting them from this process [81]. As a result, phage genomes lacking recognisable integrases, carrying their own packaging enzymes, and having structural genes would actually be able to complete their life cycle without the aid of a helper phage, and would thus be the simplest kind of autonomous phage [82]. *Bacillus* phage FI represents a novel phage amongst identified *Bacillus* phages, sequenced alongside with the bacterial host of *K. variicola*. The GC content of this phage (35.06%) is different from the *K. variicola* GC content (53.31%) and similar to the *Bacillus cereus* host. Despite *Bacillus* phage FI grouping closely with the *B. anthracis* host phages, Gamma, cherry, Wbeta and Fah phages, it displays a distinct cluster or different species (ANI < 95%) among the compared Bacillota host phages with a high number of CDS (*n* = 145). Most annotated genes (*n* = 104) were assigned with unknown function that are worth to be further investigated.

## Conclusion

This study presents a complete genome sequence of a multidrug-resistant *K. variicola* strain T2 that consists of a concurrent novel *Bacillus* phage that shows a non-deficient integrase lifestyle. Furthermore, the *Bacillus* phage GC content is highly influenced by its host, as demonstrated in this study by interacting with *Bacillus cereus* rather than with *Klebsiella* host, with a GC content of 54%. The mobile genetic elements, which included the prophage regions and five plasmids, influenced the genome size. The plasmids identified in this genome are novel among *K. variicola* genomes and mostly encode genes that are important for adaptability to environmental stress from WWTPs. This is the first report of *K. variicola* isolated from WWTP influent in South Africa. The identified *Bacillus* phage will enhance our understanding on the impact of antibiotic resistance mechanisms, particularly in WWTP. This study highlights the need for ongoing genomic epidemiology surveillance of environmental *K. variicola* isolates as well as understanding the lifestyles of *K. variicola* prophages and non-deficient phages.

## Supplementary Information

Supplementary Material 1.

## Data Availability

The genome of Klebsiella variicola strain T2 has been assigned the following accession number: GenBank CP133153-CP133158, and the phage genome: OR487170.
