# Supplementary material for: Genomic insight on Klebsiella variicola isolated from wastewater treatment plant has uncovered a novel bacteriophage
Source: BMC Genomics. 2024 Oct 22;25:986. doi: 10.1186/s12864-024-10906-x (PMC11494819; doi:10.1186/s12864-024-10906-x)
Supplement: Supplementary file 1 — Supplementary Material 1. [file 12864_2024_10906_MOESM1_ESM.docx]

**Genomic insight on *Klebsiella* *variicola* isolated from wastewater treatment plant has uncovered a novel bacteriophage**

Kgaugelo E. Lekota^1^, Refilwe O. Mabeo^1^, Tsepo Ramatla^1^, Deidre A.B. Van Wyk^1^, Oriel Thekisoe, Lesego G. Molale-Tom^1^, Cornelius C. Bezuidenhout^1^

^1^ North-West University, Unit for Environmental Sciences and Management, Potchefstroom, South Africa

Corresponding author: email: [Lekota.lekota@nwu.ac.za](mailto:Lekota.lekota@nwu.ac.za)

**Supplementary Table S1**

Designed primer sequences of *Bacillus* phage FI screened among the *Klebsiella* species isolates.

| **Primer name** | **5’-3’** | **Tm** | **Product size** | **Target coding sequence region** |
| --- | --- | --- | --- | --- |
| Primer_BF | AAACGGAATCGACAGCACCT | 60 | 536 bp | Phage terminase large subunit |
| Primer_BR | GCTTTTGCGCTTCACTTTGC | 60 |  |  |
|  |  |  |  |  |
| Primer_2BF | GCAAGCAAGTGCACTCCATA | 60 | 301 bp | Hypothetical protein |
| Primer_2BR | AGGTTCTTCATTAGACTGTGTATTACG | 60 |  |  |
|  |  |  |  |  |
| Primer_9BF | AGGCAAAAGCTGGATGGGAA | 60 | 577 bp | Tail fiber spike protein |
| Primer_9BR | TGCCCCAGAAATGCTCTAGC | 60 |  |  |

**Supplementary Table S2**

Phenotypic antibiotic resistance patterns of *Klebsiella* spp. isolated from waste water treatment plant.

| **Isolates ID** | **Resistant antibiotics (Kirby-Bauer)** | **Susceptible antibiotics** |
| --- | --- | --- |
| INF- 1A | AMP | KZ, CN, C, AM, CTX, ERT, TMP-SMZ, IMI, NFT, NOR, OFX |
| INF- 1B | AMP | KZ, CN, C, AM, CTX, ERT, TMP-SMZ, IMI, NFT, NOR, OFX |
| INF- 1C | AMP, KZ | CN, C, AM, CTX, ERT, TMP-SMZ, IMI, NFT, NOR, OFX |
| INF- 1D | AMP | KZ, CN, C, AM, CTX, ERT, TMP-SMZ, IMI, NFT, NOR, OFX |
| INF- 1E | AMP, KZ | CN, C, AM, CTX, ERT, TMP-SMZ, IMI, NFT, NOR, OFX |
| INF- 2A (strain T2) | KZ, AMP, CN, NFT | C, AM, CTX, ERT, TMP-SMZ, IMI, NFT, NOR, OFX |
| INF- 2B | AMP, KZ | CN, C, AM, CTX, ERT, TMP-SMZ, IMI, NFT, NOR, OFX |
| INF- 2C | AMP, KZ | CN, C, AM, CTX, ERT, TMP-SMZ, IMI, NFT, NOR, OFX |
| INF- 2D | AMP, KZ | CN, C, AM, CTX, ERT, TMP-SMZ, IMI, NFT, NOR, OFX |
| INF- 2E | AMP, KZ | CN, C, AM, CTX, ERT, TMP-SMZ, IMI, NFT, NOR, OFX |
| EFF- 1A | AMP | KZ, CN, C, AM, CTX, ERT, TMP-SMZ, IMI, NFT, NOR, OFX |
| EFF- 1B | AMP, KZ | CN, C, AM, CTX, ERT, TMP-SMZ, IMI, NFT, NOR, OFX |
| EFF- 1C | AMP, CN, KZ, C | AM, CTX, ERT, TMP-SMZ, IMI, NFT, NOR, OFX |
| EFF- 1D | AMP, CN, KZ | C, AM, CTX, ERT, TMP-SMZ, IMI, NFT, NOR, OFX |

INF: Influent, EFF: Effluent, AMP: Ampicillin (10µg), KZ: Cephazolin (30µg), C: Chloramphenicol (30µg), CN: Gentamycin (10μg), AM: Amikacin (30µg), CTX: Cefotaxime (30µg), ERT: Ceftriaxone (30µg), TMP-SMZ: Cotrimoxazole (25µg), IMI: Imipenem (10µg), NFT: Nitrofurantoin (300µg), NOR: Norfloxacin (10µg), OFX: Ofloxacin (5µg), γ: Gamma hemolytic, α: Alpha hemolytic.

**Supplementary Table S3**

Functional prediction of the *Bacillus* phage FI with the two compared WBeta *Bacillus* phages.

|  | *Bacillus* phage Cherry | *Bacillus* phage Gamma | *Bacillus* phage FI |
| --- | --- | --- | --- |
| **Description** | Gene count | Gene count | Gene count |
| **CDS** | 59 | 63 | 145 |
| **connector** | 4 | 4 | 3 |
| **DNA, RNA and nucleotide metabolism** | 6 | 6 | 7 |
| **head and packaging** | 6 | 6 | 11 |
| **integration and excision** | 1 | 1 | 0 |
| **lysis** | 2 | 2 | 3 |
| **moron, auxiliary metabolic gene and host takeover** | 1 | 1 | 2 |
| **other** | 0 | 0 | 0 |
| **tail** | 3 | 3 | 9 |
| **transcription regulation** | 3 | 4 | 6 |
| **unknown function** | 33 | 36 | 104 |
| **tRNAs** | 0 | 0 | 0 |
| **CRISPRs** | 0 | 0 | 0 |
| **tmRNAs** | 0 | 0 | 0 |
| **VFDB Virulence Factors** | 0 | 0 | 0 |
| **CARD AMR Genes** | 0 | 0 | 0 |
| **AMR Finder** | 0 | 0 | 1 |

**Supplementary Table S4:** Antibiotic resistance profile of *Klebsiella variicola* strain T2 identified using AMPFinder Plus

| Accession number | Gene symbol | Sequence name | Element type | Class | % Coverage of reference sequence | % Identity to reference sequence |
| --- | --- | --- | --- | --- | --- | --- |
| CP133153 | *oqxA* | multidrug efflux RND transporter periplasmic adaptor subunit OqxA | AMR | PHENICOL/QUINOLONE | 98.98 | 98.19 |
| CP133153 | *oqxB* | multidrug efflux RND transporter permease subunit OqxB | AMR | PHENICOL/QUINOLONE | 60.67 | 90.50 |
| CP133153 | *emrD* | multidrug efflux MFS transporter EmrD | AMR | EFFLUX | 100.00 | 99.24 |
| CP133153 | *fosA* | FosA5 family fosfomycin resistance glutathione transferase | AMR | FOSFOMYCIN | 100.00 | 99.28 |
| CP133153 | *kdeA* | multidrug efflux MFS transporter KdeA | AMR | EFFLUX | 96.59 | 99.24 |
| CP133153 | *Bla-LEN* | LEN family class A beta-lactamase | AMR | BETA-LACTAM | 91.96 | 100.00 |
| OR487170 | *fosB* | FosB/FosD family fosfomycin resistance bacillithiol transferase | AMR | FOSFOMYCIN | 90.65 | 92.06 |
| CP133157 | *kefB-GI* | heat resistance system K+/H+ antiporter KefB-GI | STRESS | NA | 57.79 | 97.58 |
| CP133157 | *trxLHR* | heat resistance system thioredoxin Trx-GI | STRESS | NA | 100.00 | 98.63 |
| CP133157 | *hdeD-GI* | heat resistance membrane protein HdeD-GI | STRESS | NA | 100.00 | 98.03 |
| CP133157 | *yfdX2* | heat resistance protein YfdX2 | STRESS | NA | 74.58 | 97.73 |
| CP133157 | *yfdX1* | heat resistance protein YfdX1 | STRESS | NA | 88.82 | 97.41 |
| CP133157 | *shsP* | small heat shock protein sHSP20-GI | STRESS | NA | 55.92 | 96.47 |
| CP133157 | *hsp20* | small heat shock protein sHSP20 | STRESS | NA | 100.00 | 97.88 |
| CP133157 | *silE* | silver-binding protein SilE | STRESS | SILVER | 100.00 | 88.11 |
| CP133157 | *silR* | copper/silver response regulator transcription factor SilR | STRESS | COPPER/SILVER | 100.00 | 99.56 |
| CP133157 | *silC* | Cu(+)/Ag(+) efflux RND transporter outer membrane channel SilC | STRESS | COPPER/SILVER | 67.03 | 98.38 |
| CP133157 | *silB* | Cu(+)/Ag(+) efflux RND transporter periplasmic adaptor subunit SilB | STRESS | COPPER/SILVER | 91.16 | 98.21 |
| CP133157 | *silA* | Cu(+)/Ag(+) efflux RND transporter permease subunit SilA | STRESS | COPPER/SILVER | 57.73 | 99.17 |
| CP133157 | *silP* | Ag(+)-translocating P-type ATPase SilP | STRESS | SILVER | 99.64 | 94.41 |
| CP133157 | *pcoB* | copper-binding protein PcoB | STRESS | COPPER | 100.00 | 100.00 |
| CP133157 | *pcoC* | copper resistance system metallochaperone PcoC | STRESS | COPPER | 100.00 | 100.00 |
| CP133157 | *pcoD* | copper resistance inner membrane protein PcoD | STRESS | COPPER | 55.34 | 98.25 |
| CP133157 | *pcoR* | copper response regulator transcription factor PcoR | STRESS | COPPER | 100.00 | 100.00 |
| CP133157 | *pcoS* | copper resistance membrane spanning protein PcoS | STRESS | COPPER | 100.00 | 99.14 |
| CP133157 | *terD* | tellurium resistance membrane protein TerD | STRESS | TELLURIUM | 100.00 | 98.96 |
| CP133157 | *terC* | tellurium resistance membrane protein TerC | STRESS | TELLURIUM | 83.24 | 98.96 |
| CP133157 | *terB* | tellurium resistance membrane protein TerB | STRESS | TELLURIUM | 100.00 | 98.68 |
| CP133153 | *fieF* | CDF family cation-efflux transporter FieF | STRESS | NA | 99.00 | 99.33 |


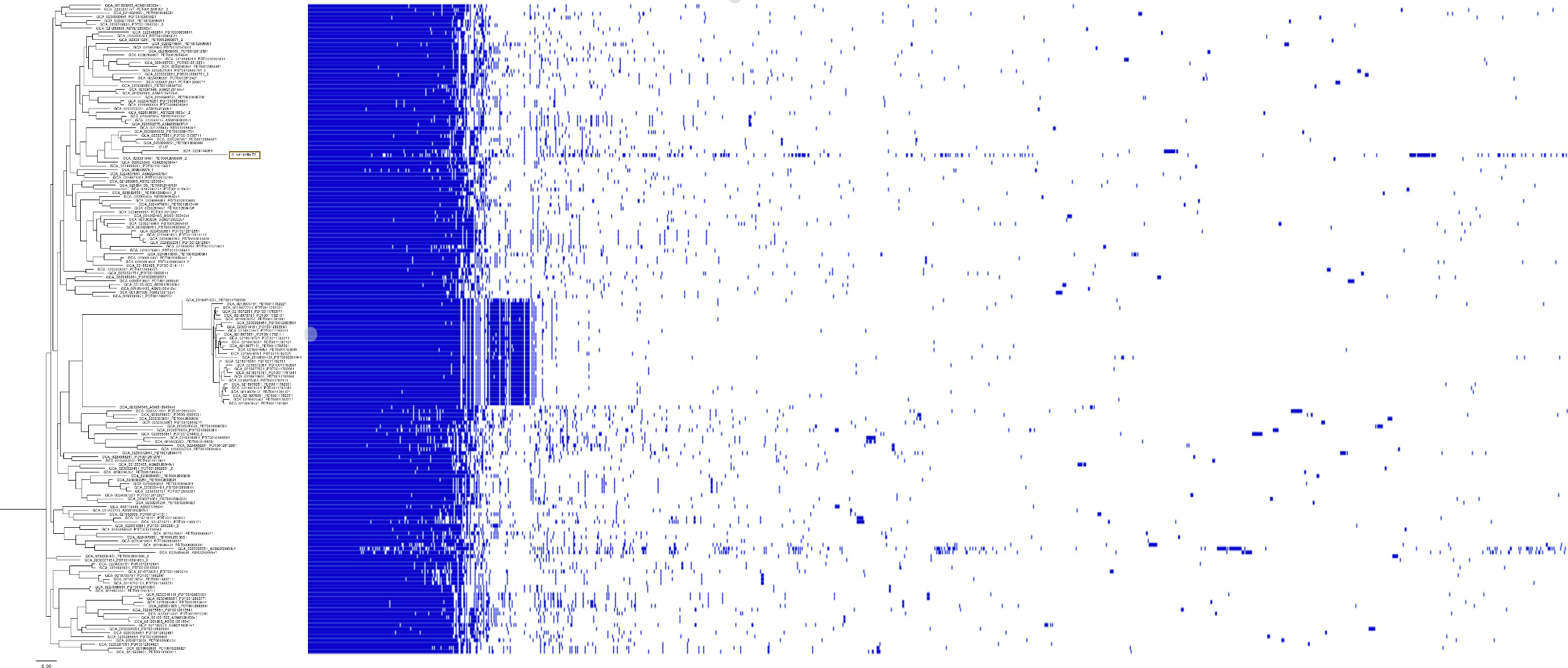
**Supplementary Fig. S1**. Core genome phylogeny of *Klebsiella variicola* from the sequenced genome and GenBank alongside a matrix of gene content. Clear delineation of the sequenced strain T2 is highlighted by a box also showing core and differences accessory gene content between the species. Pangenome analysis performed using Roary and visualization with Phandango.

**Supplementary Fig. S2.** The pan-genome profile of the closely related genomes of *K. variicola* with the sequenced strain T2 in this study. The pan genome of core gene and unique cluster are presented on top, while the number of genes clusters shared and unique amongst compared genomes are presented in the bottom. A total of 3216 unique gene clusters were found in the *K. variicola* strain T2, meanwhile while 100 orthologs gene clusters are noted between GC00875025 strain 171J7 strain and the sequenced genome in this study.

**Supplementary Fig. S3.** Circular plots of the plasmids sequenced in this study with the synteny of the compared closest related plasmids. **A**. Circular plots showing the coding sequences, and mobile genetic elements. B. Multiple sequence alignment of the closest related plasmids showing the synteny identified in this study.

**Supplementary Fig S4.** Determination of the prophage regions on the complete chromosome of *Klebsiella variicola* strain T2**.** **A.** Circular plot indicating the placement of the 5 phages identified using VirSorter i.e., 4 phages were characterized as intact or complete (phage 0, 1, 2 and 3 phage), and phage 4 as an incomplete phage using PHASTER localized from the outer first ring. Second and third ring represent GC content and GC skew, respectively. **B**. Whole protein phylogeny placement of the 5 identified prophages among Pseudomonadota host phages constructed using 74 sequences. The identified phages in this study are noted with red branches.

**Supplementary Fig. S5.** Whole genome nucleotide similarity among WBeta phages (n =19) with *Bacillus* phage FI based on ≥ 70% intergenomic similarity to cluster. Intergenomic similarities are shown on top/right hand side (scale from 0–100%) and aligned genome fraction (0–1) and genome length ratio (0–1) are shown on top together with total genome size

**Supplementary Fig. S6.** Phylogenetic placement of the sequenced *Bacillus phage KG-Lek*. Whole genome-based phylogeny (nucleotide sequence) was inferred using Genome-BLAST Distance Phylogeny (100 bootstrap) using formula D6 yielding average support of 70%. The phylogeny was constructed using amongst 16 *Bacillus cereus* group and *Klebsiella* phages. Scale bar represents the number of nucleotide substitutions per site.
